# Electrophysiological Correlation Underlying the Effects of Music Preference on the Prefrontal Cortex Using a Brain–Computer Interface

**DOI:** 10.3390/s21062161

**Published:** 2021-03-19

**Authors:** Kevin C. Tseng

**Affiliations:** Product Design and Development Laboratory, Department of Industrial Design, National Taipei University of Technology, Taipei City 106344, Taiwan; ktseng@pddlab.org

**Keywords:** brain–computer interface, EEG, music preference, prefrontal cortex

## Abstract

This study aims to research the task of recognising brain activities in the prefrontal cortex that correspond to music at different preference levels. Since task performance regarding the effects of the subjects’ favourite music can lead to better outcomes, we focus on the physical interpretation of electroencephalography (EEG) bands underlying the preference level for music. The experiment was implemented using a continuous response digital interface for the preference classification of three types of musical stimuli. The results showed that favourite songs more significantly evoked frontal theta than did the music of low and moderate preference levels. Additionally, correlations of frontal theta with cognitive state indicated that the frontal theta is associated not only with the cognitive state but also with emotional processing. These findings demonstrate that favourite songs can have more positive effects on listeners than less favourable music and suggest that theta and lower alpha in the frontal cortex are good indicators of both cognitive state and emotion.

## 1. Introduction

Music can awaken the senses and evoke powerful emotions [1,2]. Previous studies have shown that different frequency bands of electroencephalography (EEG) from a particular cortex have different physical functions and meanings. The alpha band (8–13 Hz) represents at least three types of function: attention, motor function, and emotional processing. Alpha rhythms, originating from the occipital midline, are strongly correlated with attention and vigilance, and alpha rhythms from the somatosensory cortex have been linked with movement. Moreover, Tsang et al. found that alpha power from the left frontal cortex decreased when subjects listened to positive music, as did alpha power from the right frontal cortex when listening to negative music [3]. Therefore, based on these characteristics of EEG, the present study employed an experimental paradigm to interpret the influence of music at different levels of preference using EEG activity in a psychological and physiological manner.

Two important factors in the experiment must be clearly defined. First, individuals’ objective preferences for musical genres vary greatly. Undoubtedly, music enables listeners to experience positive effects, not only on psychological consciousness but also on physical activities. These effects have been experienced in many domains, such as music therapy [4], physical rehabilitation [5], and work performance [6]. Second, to understand music’s effects on cognitive controls and emotional processing, the prefrontal cortex (PFC) is highly suitable because it includes cognitive capacities, such as learning, decision-making, execution, and emotional processing.

To date, an increasing number of studies have used EEG to interpret human behaviours and have attempted to understand the relationships between activities in various EEG frequency bands. However, little attention has been paid to the correlation between EEG responses and listening to one’s favourite music, and using EEG to investigate the links between music and psychological responses and to assess psychological characteristics remains in the developmental stage [7]. As far as we are concerned, different people with the same conditions will experience different feedback. It is a formidable task to find general agreement on musical genre taxonomies. Nevertheless, some studies have applied tempo, rhythm, and types to classify music [8,9]; at the same time, other studies have used subjective preferences as criteria for music selection [10]. Therefore, one of the aims of this study is to scientifically investigate music-elicited effects on listeners, such as higher cognitive state, attention, and positive emotions.

Consequently, this study aims to apply an objective EEG response to investigate whether an individual’s preference for music would significantly enhance brain activities in the PFC. To investigate both psychological and physiological feedback according to music, we first hypothesised that EEG power from the PFC would respond more strongly if subjects listened to their favourite music rather than other music. Second, the left and right PFC would demonstrate alpha power with favourite music due to the hemispheric asymmetry hypothesis [11]. We assumed that the study would reveal that EEG on the left PFC would be elicited by the stimulus of favourite music, while decay would occur in the right PFC. Therefore, this study applied a real-time brain–computer interface (BCI) system to retrieve and analyse EEG responses in different situations and used a continuous response digital interface (CRDI) [12] to record people’s feelings regarding musical stimuli in real-time [13,14,15].

## 2. Materials

**Figure 1:**
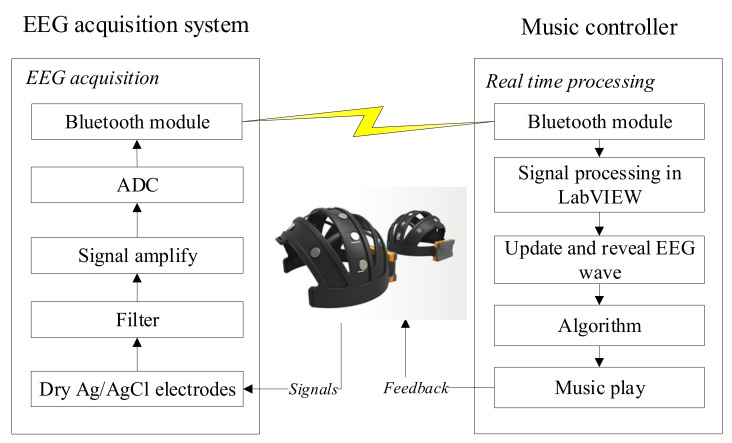
The overall framework of the proposed brain–computer interface (BCI) music controller.

The overall framework of the proposed BCI-based music controller system is shown in Figure 1. The proposed system included an EEG acquisition module, a Bluetooth module, a real-time analysis module, and a music controller to control music playback according to the subject’s brain waves and to enhance the effect of music therapy. The current music is recommended based on the subject’s preference, but when the subject’s mood changes (such as happiness, anger, sadness), the designed system can recommend songs that match the subject’s current mood and preference to make him/her feel happier. Therefore, the proposed system can acquire EEG signals synchronously and transmit these EEG signals to a backend system for further analysis. A music control program was written in the C# programming language for the management of the experimental procedure, and three types of musical stimuli were used in this study.

### 2.1. EEG Acquisition Module

**Figure 2:**
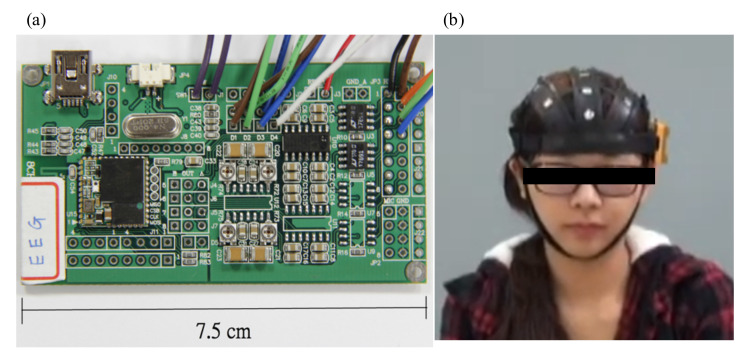
Photographs of (**a**) our EEG acquisition module and (**b**) the EEG cap embedded with the module.

The EEG acquisition module was developed with the capability for continuous low-power operation for more than 30 h. This module contained a microprocessor (TI MSP430), which provided an 8-channel 12-bit successive approximation register (SAR) analogue-to-digital conversion (ADC) and a 256-Hz sampling rate, a Bluetooth transmitter circuit and a front-end amplifier. The Bluetooth transmitter circuit contained a Bluetooth module, compliant with v2.0+ EDR specifications, and a printed circuit board antenna. The microprocessor communicates with the Bluetooth module through a universal asynchronous receiver/transmitter (UART). The front-end amplifier consists of two parts: an instrument amplifier and a high-pass filter. The amplifier filters and amplifies EEG signals acquired from the designed EEG electrodes. The gain of the amplifier was 5000 times the frequency band of more than 0.1 Hz. To miniaturise the size of the EEG acquisition module, a moving-average filter, operating in the microprocessor, was designed to digitise EEG signals, perform low-pass filtering, control the peripheral circuits, and send EEG data to the Bluetooth module. The indicative cut-off frequency is a moving average of 56 Hz to reduce the power-line interference and noise at a higher frequency prior to sending EEG signals to the Bluetooth module. The size of the EEG acquisition module is approximately 75 × 45 × 6 mm, and it can be embedded into the designed EEG cap as a wearable device, as shown in Figure 2.

### 2.2. Stimuli

This study is based on music’s melody and tempo when choosing three different types of music: subjects’ favourite song (FS), Mozart K.448 (K448), and high focus music (HF). Three categories of music were randomly presented to the subjects for testing of preference levels and brain activity. This study applied FS selected by the subjects, K448 for two pianos in D major (a famous concerto composed by Mozart that has been popularly used as stimuli in many studies [16,17]), and Track 2 of HF blends, with ambient sounds with 40-Hz beta waves for the experiment. The reason to blend HF with ambient sounds with 40-Hz beta waves is that beta waves are high-frequency, low-amplitude brain waves commonly observed in an awakened state. Generally, high beta waves (18–40 Hz) are associated with significant stress, anxiety, paranoia, high energy, and high arousal [18]. Nevertheless, the CRDI was used to define the subjects’ preference level for music and to help them select suitable favourite songs. The two-dimensional CRDI system is designed to collect continuous self-reported data in real-time while the subject is experiencing the stimulus.

## 3. Methods

To evaluate the impact of the stimuli on the listeners and EEG variations of the effects during the time sequence of music, the proposed BCI-based music controller was used in the experiment.

### 3.1. Subjects

The study was conducted according to the guidelines of the Declaration of Helsinki and approved by the Institutional Review Board of Chang Gnug Medical Foundation (protocol code 201601957B0C501 and date of approval: 27 October 2020). Twenty-eight subjects (13 of 28 subjects were male, and the mean age was 21.75 years old) enrolled in the experiment. Informed consent was obtained from all of the subjects involved in the study. None of the subjects had any formal musical training or any experience playing instruments in order to minimise the influence of professional musical knowledge on the stimuli. Additionally, none of them reported hearing problems or neurological diseases.

### 3.2. Experimental Design

The experiment comprised five silent breaks (1-min intervals of silence) between each stimulus. Before the experiment, the subjects listened to HF and K448 in advance to ensure that unfamiliarity with the stimuli would not influence the subjective preference level. At the beginning of the experiment, the staff introduced the subjects to the purposes of the study, instructed them to complete the questionnaire on their self-rated cognitive state, and ensured that the sleeping hours of each subject were more than 6 h. The questionnaire assesses an individual’s current state level on a scale from 1 (drowsiness) to 10 (wakefulness). During the course of the experiment, the subject stood in a soundproof room and sat in a chair, which minimised environmental noise and possible interference. During the trial, the subjects could relax with a 1-min silent period of preparation, which was omitted to avoid negative effects on the baseline. The baseline consisted of a 1-min silent period for normalisation. The other intervals between the stimuli prevented the participants from interfering with the music. After the end of the experiment, the subjects were asked to assess their state level during each stimulus using the same scale.

### 3.3. EEG Recordings, Data Processing, and Statistical Analysis

According to the scheme of the proposed BCI-based music controller, shown in Figure 1, EEG was recorded with five dry Ag/AgCl electrodes placed on the frontal lobe. F_pz_, located on the forehead, was used as a ground electrode, and the left mastoid was used as a reference. This experiment intended to investigate the effects of the three provided types of music on emotion processing and cognitive state. The others were placed at F_p1_, F_p2_, and F_z_ on the PFC, representing mental activities, sustained attention, and emotional processes, respectively [19,20]. Furthermore, beta waves indicate alertness, cognitive processes, and motor functions. The signals were processed by the EEG acquisition system, with a sampling rate of 256 Hz. A 256-point fast Fourier transformation (FFT), with 50% overlap, was used to calculate the mean power of the EEG power spectrum. The Hanning window function in LabVIEW software was implemented before FFT to reduce spectral leakage. The EEG frequency spectrum was defined by calculating the EEG mean power spectrum and dividing it into theta (4–7 Hz), alpha (8–13 Hz), and beta (14–40 Hz) bands.

The results showed that when the relation ratio (RR) value increased, the participant was prone to positive emotions and vice versa. In the statistical analysis, two-way ANOVA with factors of positions (F_p1_, F_p2_, and F_z_) and time (HF, K448, and FS) was applied to obtain the altered effects in the response from the PFC.

## 4. Results

### 4.1. Preference Ratings and Subjective Evaluation of the Cognitive State

As we assumed, music with higher preference levels had better effects on listeners. In many studies in music research, the mean and standard deviation (SD) in a whole musical piece are the two essential indices for CRDI analysis. This study represents an initial investigation utilising two-dimensional CRDI analysis to track simultaneous listener perceptions of emotional responses in an attempt to better understand how subjects’ emotions are evoked by music. During the experiment, the system automatically records the brainwave physiological response of the subject and uses CDRI to measure and record the degree of preference so that the subject can continue to record his/her preference for the current music.

However, averaging the preferred response during the overall period to a musical excerpt easily influences the correction by evening out the preferred result of the excerpt. In the experiment, many subjects’ preference curves sketched upward quickly at the beginning of a musical piece and then continued steadily upward until the end of the piece, according to the raw CRDI data, revealing the subjects’ feelings about the stimuli in real-time. Therefore, to eliminate and avoid deviation at the beginning of the measurement, we proposed a slope, rather than an average, as an index of preference intensity. Furthermore, to calculate the slope of the curve, we applied a first-degree polynomial equation to fit the preference curve of a musical piece using MATLAB software.

**Figure 3:**
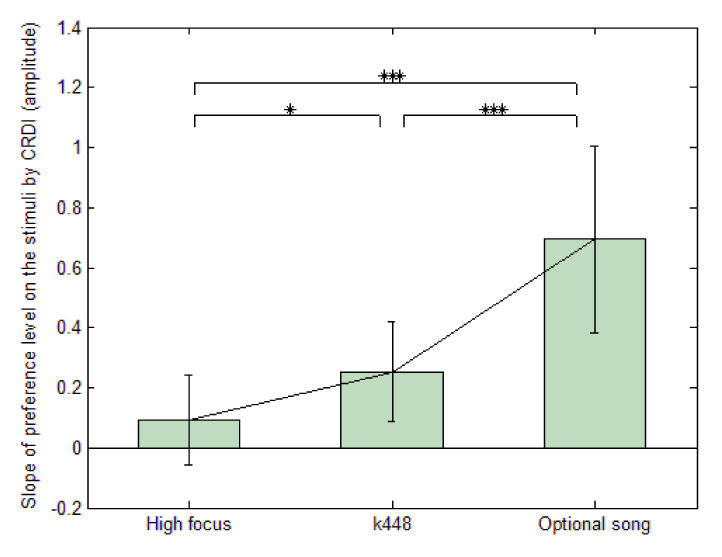
The slope and average preference level that the subjects rated for high focus music (HF), Mozart K.448 (K448), and favourite song (FS) by continuous response digital interface (CRDI), analysed by ANOVA; *: *p* < 0.05; ***: *p* < 0.001.

Figure 3 shows the intensity of the preference levels of the subject-selected favourite song, Mozart K.448, and HF music. Different from the meaning of amplitude, the higher slope represents greater intensity that the musical pieces imposed on the subjects. Therefore, considering 0.5 the median intensity by rule of thumb, FS evoked more intense and positive emotions for the subjects than HF or K448 (test values were −14.51, −7.88, and 3.29, with *p* < 0.001, *p* < 0.001, and *p* < 0.01, respectively). In addition, the intensity evoked by HF and K448 was less than the median, indicating that the preference intensity was significantly different for each stimulus, according to the analysis of the repeated-measures one-way ANOVA. This result suggests that the features of the three types of stimuli were discernible for the subjects to evoke discrete emotions.

**Figure 4:**
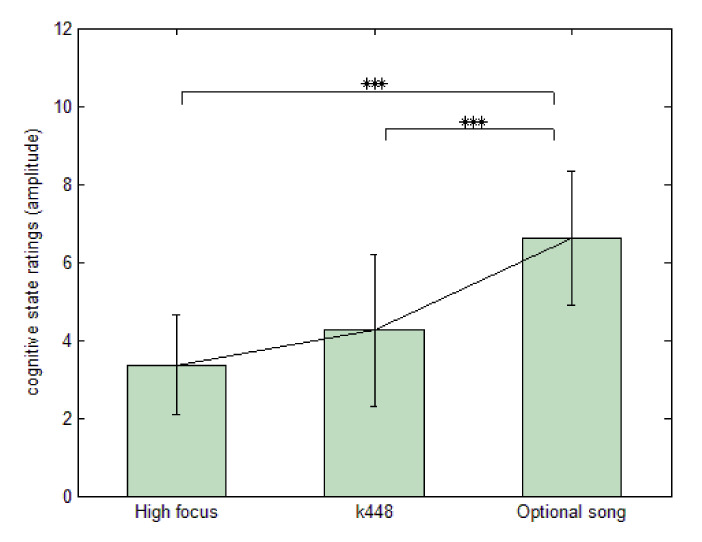
Self-rating of cognitive state, analysed by repeated-measures one-way ANOVA for independence; ***: *p* < 0.001.

**Figure 5:**
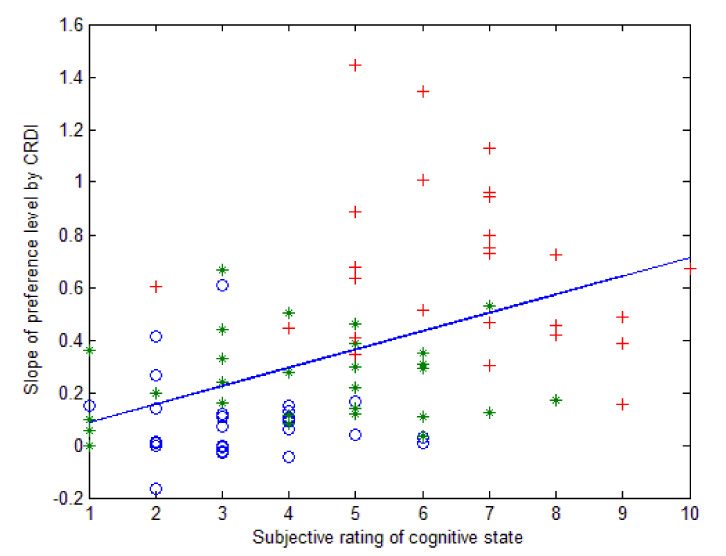
Median correlation between intensity and cognitive state, measured by Pearson’s product-moment correlation; correlation = 0.446, with *p* < 0.001; *: K.448; ○: HF music; +: subject-selected favourite song.

To research the cognitive states involved in brain activities, Figure 4 shows the results of self-rating on cognitive state stimulated by HF, K448, and FS, respectively, analysed by ANOVA (denoting M = 3.36, 4.25, and 6.61; SD = 1.28, 1.96, and 1.73). The results show that the subjects agreed that FS aroused significantly higher state levels than HF and K448. In contrast, HF and K448 evoked no comparably different states for the subjects. Furthermore, by applying Pearson’s product-moment correlation to calculate the correlation between cognitive state and CRDI response, the results revealed a significantly moderate correlation, as shown in Figure 5. This result suggests that the intensity and amplitude of the preference level were linear with the subjects’ subjective ratings of cognitive state, indicating that greater preference and intensity would positively affect the listeners’ cognitive states.

**Figure 6:**
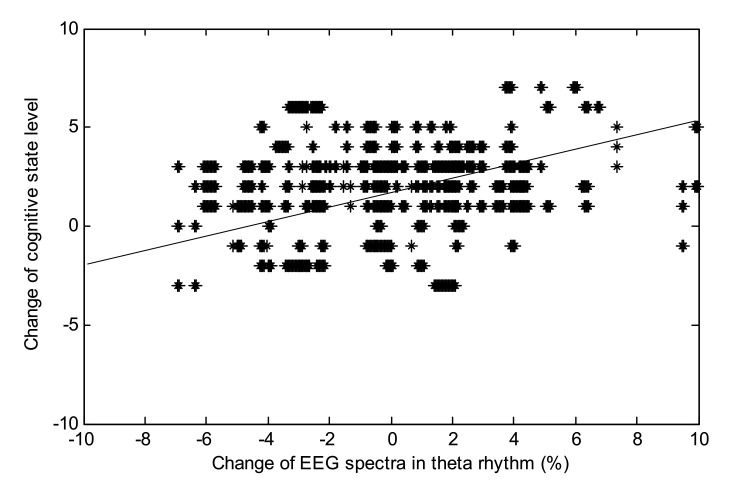
The relationship between cognitive state and theta waves.

Furthermore, according to the continuous self-report results of the subjects’ cognitive state, the response of the prefrontal theta wave was compared, as shown in Figure 6. As previously expected, the prefrontal theta waves of most subjects were in line with the trends of their self-reporting results. This result indicates that as the subject’s self-reported cognitive state gradually increases with the song, the theta wave also improves, objectively showing that the subject’s cognitive state is gradually improving. At the same time, it also said that the more one likes music, the more significant its effect on brainwaves is.

### 4.2. Statistical Analysis for EEG Waves

The primary focus of the current study is the use of theta, alpha, and beta rhythms to explore the activation of sensory cortices during experiments and observations [21,22,23]. This study performed separate analyses on theta (4–7 Hz), alpha (8–13 Hz), lower alpha (8–10 Hz), upper alpha (11–13 Hz), and beta (14–40 Hz) bands. To determine how EEG waves from the different positions on the scalp were affected by different types of music, position and time (defined by ANOVA) accounted for the electrode positions and the sequence of playing the stimuli, respectively. Table 1 shows the ANOVA results, with factors and the interaction of the factors regarding F-value, *p*-value, and effect size. These outcomes suggest that the more intensely favourited that the music was, the greater the arousal of brain activity at the PFC, while EEG power revealed more noticeable increments with higher preference levels. Additionally, the results showed that theta and lower alpha power had significant effects towards the end of the experiment, according to ANOVA with the factor of time. Upper alpha and beta power, however, did not reach statistical significance, although they were both increased on average. In contrast, upper alpha and beta power were affected significantly by the factors of position (Fz, Fp1, and Fp2). Interestingly, there was no significance in the interaction between the factors of position and time, indicating that temporal and spatial factors were independent of each other. Nevertheless, time positively influenced the EEG waves more than position.

Based on ANOVA, the time factor confirmed that theta, lower alpha, and alpha at the PFC were significantly increased towards the end of the stimuli, whereas the same effects on upper alpha and beta were observed by the main factor of position because time and position are independent of each other. To further investigate the EEG response at the three positions, the paired-sample t-test was used to measure the changes in normalised EEG bands compared to the baseline of each band with the factor of time. The results, shown in Table 2, revealed that theta and lower alpha power from the PFC was significantly increased compared to baseline during the period of FS, while no significance was found with HF or K448. Compared with other EEG bands at the three positions, upper alpha at Fp1 received better effects from HF and FS but the opposite results at Fz and no significant response at Fp2. For the beta band, a significant rise in Fz during FS was observed. As the author expected, the results were well supported by ANOVA, using the factor of time.

Table 3 shows the EEG correlation with CRDI results and the subjective rating of cognitive state. Theta power from the PFC was low to moderate and was significantly correlated with the cognitive state and emotional response of the subjects. Listening to FS gradually affected the subjects, arousing their cognitive state and becoming pleasant. However, only lower alpha, upper alpha, and beta power at Fp2 had significant low-to-moderate correlations with cognitive state. No similar results with intensity at other positions were found.

## 5. Discussion

The present study aimed to determine the brain mechanism of the PFC underlying stimulation by music with different preference levels. The study researched the influences induced by HF, K448, and FS on brain activity, as well as the interaction of these activities, using subjective self-reporting of cognitive state and emotion.

### 5.1. Behavioural Rating

The behavioural rating indicated that the three types of stimuli induced the subjects’ distinct levels of preference for each stimulus. Differing from the average over the entire period of musical excerpts, which caused preference levels to be lower than expected, intensity revealed a better trend towards the preference level. Such ratings fit well with self-reports of cognitive state. In particular, higher preference intensity could evoke a higher arousal state, as well as stronger emotions. Additionally, these results suggest that HF and K488 evoked similar effects on the subjects, although HF and K448 evoked significant differences in preference levels because no comparable differences in stimulation or self-rating of the cognitive state were found. However, according to the bpm analysis, as the author expected, it was not quicker beats that better evoked the state and matched the results of preference rating, perhaps implying that good music recommendation should be integrated with musical features, which were treated as preferences in this study.

### 5.2. Theta Power and Alpha Power

In this experiment, theta, alpha, and beta power increased in the series of stimuli and reached a maximum compared to the other stimuli during the musical excerpt of FS. However, no significant increases in EEG were observed during the musical pieces of HF or K448. The results support that prefrontal theta power plays an important role in emotion. However, unlike research revealing an increase in theta during unpleasant music and pleasant music, no comparable power enhancements were observed during musical excerpts labelled as neutral and moderate preferences. This finding implies that the music for which people have strong preferences, both positive and negative, rather than neutral music, causes obvious effects to occur. Moreover, theta power in the PFC might also be related to attention when compared and correlated with the subjective cognitive state of the subjects. In other words, it is conceivable that the subjects became attentive and reached higher arousal states while listening to their favourite music. This finding suggests that people are attracted to music with which they are familiar, and this keeps their attention [24]. Therefore, according to the relationships among theta, preference, and cognitive state, theta in the PFC is strongly correlated with both positive emotion and cognitive state [25].

Regarding the hemispheric asymmetry hypothesis, no significant differences were found in alpha power elicited by the stimuli in the bilateral prefrontal cortex. However, an obvious but not significant (by paired t-test) increase in the relation ratio between both hemispheres was found, revealing that subjects hearing the stimuli were prone to positive moods. These results were found in other works [19], and the phenomenon of asymmetry in the frontal alpha has also been found in many studies [26]. The possible reasons that caused these bifurcated outcomes might have been different types of stimuli, such as visual tasks, memory tasks, and game-oriented tasks, rather than musical stimuli, resulting in diverse emotional responses. Additionally, different EEG references, suggesting that the average power at the right and left mastoids was more proper for frontal asymmetry, would fail asymmetry tests. This fact was confirmed by a review [27].

### 5.3. Lower Alpha, Upper Alpha, and Beta Power

A great number of studies have shown that lower alpha is associated with alertness, vigilance, and attention [25]; at the same time, upper alpha is related to cognitive and mental processing. Similar to theta power, a significantly comparable increase in lower alpha power reflects a higher cognitive state and preference. However, the correlation results showed no strong connections between lower or upper alpha and preference. It has been suggested that lower alpha can modulate the cognitive state but not an emotional process, and it differs from theta by representing both cognitive state and emotion. In contrast, no significant differences in upper alpha power were observed, although we found that upper alpha showed a prominent increase in the left prefrontal cortex. It seems that lower alpha behaves similarly to theta in attention as well as upper alpha in mental tasks. Lower alpha might be more suitable for interpreting cognitive state than upper alpha because the findings were more relevant to the cognitive state evoked by musical stimuli. However, no comparable increase in the beta band indicated that beta in the PFC had less strong links with cognitive state or emotion, perhaps suggesting that beta in the PFC was less strongly associated with the cognitive state than beta in the occipital–parietal cortex.

## 6. Conclusions

While the collected data represent a small sample of a population, the results suggest some interesting implications for music effects and brain research. If a specific aspect, or combination of aspects, of music appears to be closely related to cognitive state and emotion perception, it might be helpful to know whether an individual’s preference in music would significantly enhance brain activities in the PFC. The study discovered the brain mechanism of the PFC, underlying stimulation by music with different preference levels. Three categories of music on brain activity were examined using subjective self-reporting of cognitive state and emotion. The outcomes of the EEG-music experiment were from the foundation of three stimuli, which caused short-term effects on the subjects. No long-term effects were discussed in this study, making it somewhat difficult to conceive and compare whether listening to one’s favourite music over a long period would still achieve the same effects or even better effects. Furthermore, most of the FS in this study was coincidentally vocal music, differing from instrumental music (e.g., K448). It is reasonable to suggest that this preference might be a general norm regarding music recommendations, according to the better results of the experiment. However, whether musical types (i.e., vocal and instrumental) or musical features (i.e., melody and tempo) play a more important role in interpreting the effects on EEG power remains unclear, although our findings might indicate that vocal music can positively evoke better states. In the future, it would be fruitful to research these two issues further.

## Figures and Tables

**Table 1:** Summary of ANOVA with factors: channel and stimuli for theta, lower alpha, upper alpha, and beta bands.

Factor(s)	Dependent Variables	*F*-Values	*p*-Values	Partial $\eta^{2}$
Position	Theta	0.748	0.474	0.006
	Alpha	1.556	0.213	0.013
	Lower alpha	1.527	0.219	0.012
	Upper alpha	5.543 **	0.004	0.044
	Beta	5.528 **	0.004	0.044
Time	Theta	10.739 ***	<0.001	0.081
	Alpha	5.930 **	0.003	0.047
	Lower alpha	5.648 **	0.004	0.044
	Upper alpha	2.924	0.056	0.023
	Beta	2.910	0.056	0.023
Position × Time	Theta	0.076	0.989	0.001
	Alpha	0.101	0.982	0.002
	Lower alpha	0.102	0.982	0.002
	Upper alpha	0.075	0.99	0.001
	Beta	0.076	0.99	0.001

Note. **: *p* < 0.01; ***: *p* < 0.001.

**Table 2:** Summary of theta, lower alpha, upper alpha, and beta power at different positions around baseline at corresponding positions using the paired-sample *t*-test.

	HF				k448				FS			
	Theta	Lower Alpha	Upper Alpha	Beta	Theta	Lower Alpha	Upper Alpha	Beta	Theta	Lower Alpha	Upper Alpha	Beta
Position: Fz												
Test value	0.01	0.056	2.517 *	0.101	−1.199	−0.908	0.52	−1.428	−2.966 **	−2.07 *	−0.435	−2.165 *
*p*	0.992	0.956	0.018	0.92	0.241	0.372	0.607	0.165	0.006	0.048	0.667	0.039
Position: Fp2												
Test value	−0.584	−0.836	−1.017	−0.882	−1.237	−1.277	−1.496	−1.42	−3.513 **	−2.427 *	−1.928	−1.903
*p*	0.564	0.441	0.318	0.386	0.227	0.212	0.146	0.167	0.002	0.022	0.064	0.068
Position: Fp1												
Test value	−0.319	−0.681	−2.246 *	−0.14	−0.903	−1.123	−2.381 *	−1.141	−3.578 **	−3.118 **	−3.326 **	−1.928
*p*	0.752	0.501	0.033	0.989	0.375	0.272	0.025	0.264	0.001	0.004	0.003	0.064

Note. *: *p* < 0.05; **: *p* < 0.01.

**Table 3:** Correlations among electroencephalography (EEG) bands, CDRI tests, and subjective ratings of cognitive state.

Positions	EEG Bands	Pearson Correlation		*p*-Values	
		CS	Intensity	CS	Intensity
Fz	Theta	0.257 *	0.227 *	0.018	0.038
	Lower alpha	0.204	0.154	0.063	0.976
	Upper alpha	0.198	−0.003	0.071	0.163
	Beta	0.198	−0.003	0.072	0.976
Fp2	Theta	0.294 **	0.291 **	0.007	0.007
	Lower alpha	0.268 *	0.211	0.014	0.858
	Upper alpha	0.254 *	−0.020	0.020	0.054
	Beta	0.254 *	−0.019	0.020	0.862
Fp1	Theta	0.271 *	0.242 *	0.013	0.027
	Lower alpha	0.168	0.158	0.127	0.915
	Upper alpha	0.128	0.012	0.244	0.151
	Beta	0.127	0.011	0.251	0.918

Note. *: *p* < 0.05; **: *p* < 0.01.

## Data Availability

No new data were created or analyzed in this study. Data sharing is not applicable to this article.
